# An *in vitro* proof-of-principle study of sonobactericide

**DOI:** 10.1038/s41598-018-21648-8

**Published:** 2018-02-21

**Authors:** Kirby R. Lattwein, Himanshu Shekhar, Willem J. B. van Wamel, Tammy Gonzalez, Andrew B. Herr, Christy K. Holland, Klazina Kooiman

**Affiliations:** 1 Department of Biomedical Engineering, Thoraxcenter, Erasmus MC, Room Ee2302, P.O. Box 2040, 3000 CA Rotterdam, The Netherlands; 2 Department of Internal Medicine, Division of Cardiovascular Health and Disease, University of Cincinnati, Cincinnati, Ohio, USA; 3 Department of Medical Microbiology and Infectious Diseases, Erasmus MC, Rotterdam, The Netherlands; 4 Cincinnati Children’s Hospital Medical Center, Division of Immunobiology, Center for Systems Immunology, and Division of Infectious Diseases, Cincinnati, Ohio, USA

## Abstract

Infective endocarditis (IE) is associated with high morbidity and mortality rates. The predominant bacteria causing IE is *Staphylococcus aureus* (*S. aureus*), which can bind to existing thrombi on heart valves and generate vegetations (biofilms). In this *in vitro* flow study, we evaluated sonobactericide as a novel strategy to treat IE, using ultrasound and an ultrasound contrast agent with or without other therapeutics. We developed a model of IE biofilm using human whole-blood clots infected with patient-derived *S. aureus* (infected clots). Histology and live-cell imaging revealed a biofilm layer of fibrin-embedded living Staphylococci around a dense erythrocyte core. Infected clots were treated under flow for 30 minutes and degradation was assessed by time-lapse microscopy imaging. Treatments consisted of either continuous plasma flow alone or with different combinations of therapeutics: oxacillin (antibiotic), recombinant tissue plasminogen activator (rt-PA; thrombolytic), intermittent continuous-wave low-frequency ultrasound (120-kHz, 0.44 MPa peak-to-peak pressure), and an ultrasound contrast agent (Definity). Infected clots exposed to the combination of oxacillin, rt-PA, ultrasound, and Definity achieved 99.3 ± 1.7% loss, which was greater than the other treatment arms. Effluent size measurements suggested low likelihood of emboli formation. These results support the continued investigation of sonobactericide as a therapeutic strategy for IE.

## Introduction

Infective endocarditis (IE) is a life-threatening microbial infection of the heart valves and surrounding tissue, including endocardial prosthetic material. IE is associated with high morbidity and mortality (15–40% in-hospital and 40–69% 5-year mortality)^1–4^. Current standard treatment for IE consists of prolonged, intensive intravenous antibiotic therapy^2^.

IE is characterized by valvular vegetations (biofilms) composed primarily of a thrombus-like mesh consisting of platelets, fibrin, extracellular polymeric substance, and bacteria at different stages of replication^5,6^. Staphylococcal, streptococcal, and enterococcal species of bacteria have been implicated as the primary cause of IE^1,2^. *Staphylococcus aureus* (*S. aureus*) has been reported to have the single highest prevalence (30–31%) in IE^1^ and is associated with the highest mortality and worst prognosis. These bacteria initiate colonization by adhering to microthrombi present on valves, caused either by endothelial inflammation, mechanical damage, or spontaneous formation on intact valvular surfaces^1,5,7^.

High-risk surgical procedures may be required to treat IE, but this treatment is contraindicated in a large population of patients^3^. The presence of bacterial biofilm makes treatment challenging due to increased resistance to antibiotic action and the presence of bacteria in a dormant metabolic state. Bacteria situated within biofilms can be 100–1,000-fold less susceptible to antibiotics than the planktonic bacteria released from biofilms^8,9^. Furthermore, prolonged, high-dose antibiotic therapy paradoxically preserves persister cells within a biofilm that are tolerant to antibiotics^9^. When antibiotic therapy has concluded, persisters can switch phenotype and produce new biofilm, thereby reinitiating infection^9^. Adjuvant therapies for IE are desperately needed.

To treat various bacteria, both planktonic and residing in biofilms, other groups have successfully used low-frequency ultrasound (US) [≤1 MHz] combined with antibiotics^10–14^. Acoustic cavitation and streaming have been identified as the dominant mechanisms for what appears as an increase in antibiotic efficacy and penetration into biofilms. However, these studies have employed high acoustic pressures to induce inertial cavitation, which could induce undesirable bioeffects^15^. Using ultrasound contrast agents (UCAs) as cavitation nuclei reduces the acoustic pressure threshold for producing cavitation^16^, which could help translate this therapy to the clinic. UCAs are composed of encapsulated gas microbubbles (MBs; 1–10 μm in diameter) that oscillate volumetrically in response to US pressure variations, a phenomenon known as acoustic cavitation^17^. Cavitation of MBs has been shown to enhance US-induced bioeffects, which include drug delivery, cell death, and dissolution of thrombi (sonothrombolysis), by mechanisms such as enhanced fluid transport, sonoporation, and stimulated endocytosis^17–20^. Other investigators have tested the use of US in combination with MBs for enhancing biofilm degradation^21–24^. However, these studies have not evaluated the potential to enhance treatment of IE biofilms either by US, or US with UCAs.

In this paper, we report the results of a translatable, proof-of-principle study for treating *S. aureus* IE biofilms in an *in vitro* flow model. This treatment strategy that we have termed *sonobactericide*, combines US exposure and an UCA, with or without antibiotics or other therapeutics, to treat IE infections. A therapeutic of interest could be recombinant tissue plasminogen activator (rt-PA), a thrombolytic agent, because successful treatment of paediatric patients with IE has been reported previously^25–28^. We hypothesized that combination treatment with an antibiotic and a thrombolytic, in the presence of US and UCAs, will enhance the efficacy of *S. aureus* IE biofilm treatment. To test this hypothesis *in vitro*, we developed an infected clot model using *S. aureus* from an IE patient and human whole blood to replicate the *in vivo* early pathogenesis of IE. Infected clots were treated over a 30-minute period using an *in vitro* flow model equipped with time-lapse microscopy^29,30^. Infected clots were exposed to continuous human plasma flow either alone or with different combinations of the following: oxacillin (the antibiotic used clinically for the treatment of staphylococcal IE infections^31,32^), rt-PA, UCA Definity, and intermittent continuous-wave US. The cavitation activity nucleated by UCA was monitored using a passive detector. Treatment efficacy was assessed by measuring the infected clot width loss by bright-field microscopy. In addition, the particle size profile of the effluent produced by treatment was characterized to assess the likelihood of emboli formation.

## Results

### Bacteria isolate characterization

The *S. aureus* IE clinical isolate used in this study was found to have the *spa*-type t021. Oxacillin susceptibility was determined to be less than 2 μg/mL, thereby classifying it as methicillin-susceptible. The *S. epidermidis* quality control strain was resistant to all oxacillin concentrations, which is in accordance to its already well-known methicillin-resistant status. A growth curve indicating the lag, exponential, and stationary phase was completed for the *S. aureus* IE isolate in Iscove’s Modified Dulbecco’s Medium (IMDM). Based on this curve, all inoculums were prepared for experiments when bacteria were in the mid-exponential growth phase, around an optical density at 600 nm (OD_600nm_) of 1.

### Histological analysis and confocal viability assay of infected clots

**Figure 1:**
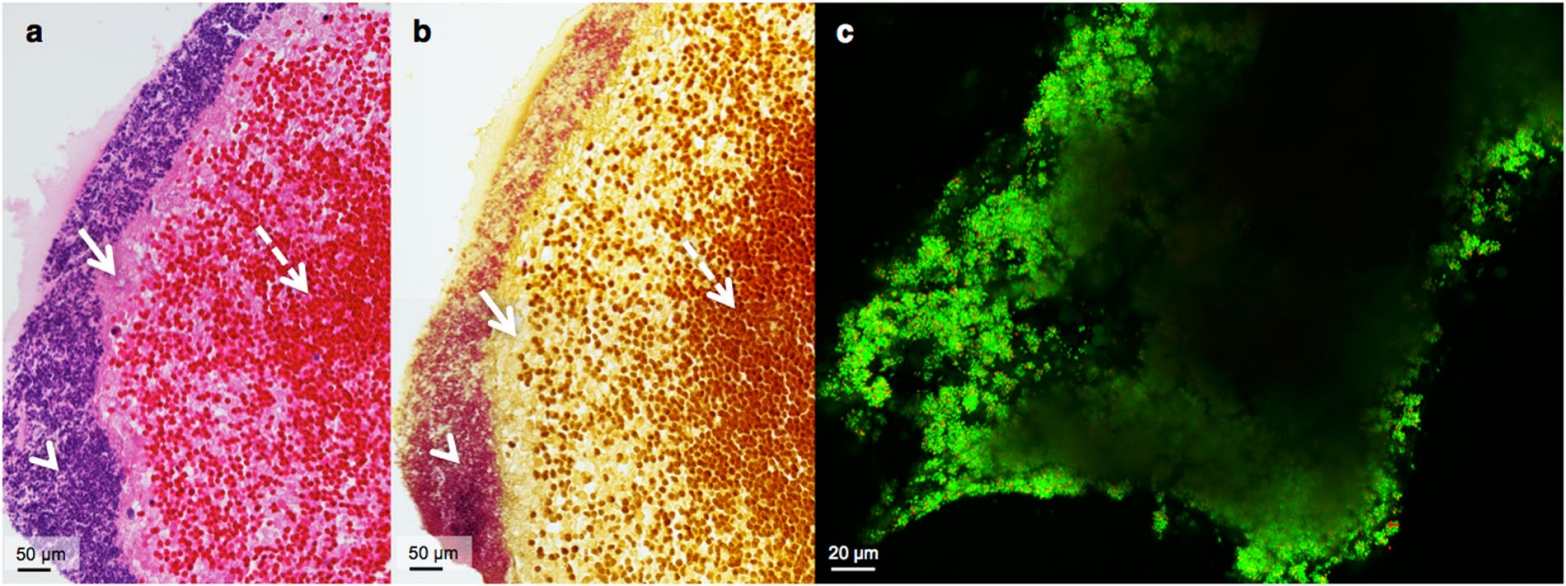
Cross-sectional histological staining and confocal laser scanning microscopy of the infected clot model. Both **a** and **b** are each composed of two combined microscope images acquired at 40x magnification. (**a**) Image of H&E staining of an infected clot cross-section. The arrowhead indicates bacteria (purple), the arrow points at fibrin (pink), and a dashed-line arrow at erythrocytes (red). (**b**) Crystal violet staining, where an arrowhead indicates bacteria (purple), an arrow for fibrin (yellow), and a dashed-line arrow for erythrocytes (brown). (**c**) ImageJ maximum intensity projection of live (green, SYTO 9 stained) and dead (red, PI stained) *S. aureus* comprising the outer layer of a representative infected clot.

Close inspection of the infected clots at high magnification showed a biofilm outer layer consisting of fibrin-embedded *Staphylococci* (Fig. 1a,b). Directly below the dense matrix of large quantities of bacteria within the fibrin mesh was a layer of fibrin. The inner portions of the clots were comprised of fibrin, sporadic immune cells, and predominately erythrocytes. Additionally, locations further from the clot core contained fewer erythrocytes, and an increasing amount of fibrin, seen in both the haematoxylin-eosin (H&E) and crystal violet (CV) staining (Fig. 1a,b). CV staining confirmed the location and presence of staphylococcal bacteria, because on a cellular level only gram-positive bacteria are stained purple (Fig. 1b). Of additional note, the iodine in the CV resulted in the staining of the erythrocytes brown and the fibrin yellow.

The interpretation of the living status of the infected clot bacteria could not be obtained from H&E and CV histological staining. Therefore, confocal laser scanning microscopy (CLSM) with two fluorescent markers was used to determine if bacteria were viable before flow experiments. In each case, the biofilms lining the clots were made up of predominately viable bacteria of 0.5–1 *μ*m spheres (-cocci) (see Fig. 1c; fluorescently labelled green by Syto 9). Dead bacteria (fluorescently labelled red by propidium iodide) were present, however at substantially lower numbers than viable bacteria. Corresponding to the histology, the thickness of the biofilms was not homogeneous. Additionally, the biofilm structure did not appear completely intact in some places (e.g. bottom left corner, Fig. 1c). Occasionally observed, most likely due to green auto-fluorescence, were rod-shaped fibre-like chains, suggesting fibrin, and round objects larger than bacteria, suggesting erythrocytes, immune cells, or possibly platelets.

### Histological comparison of infected and control clots

**Figure 2:**
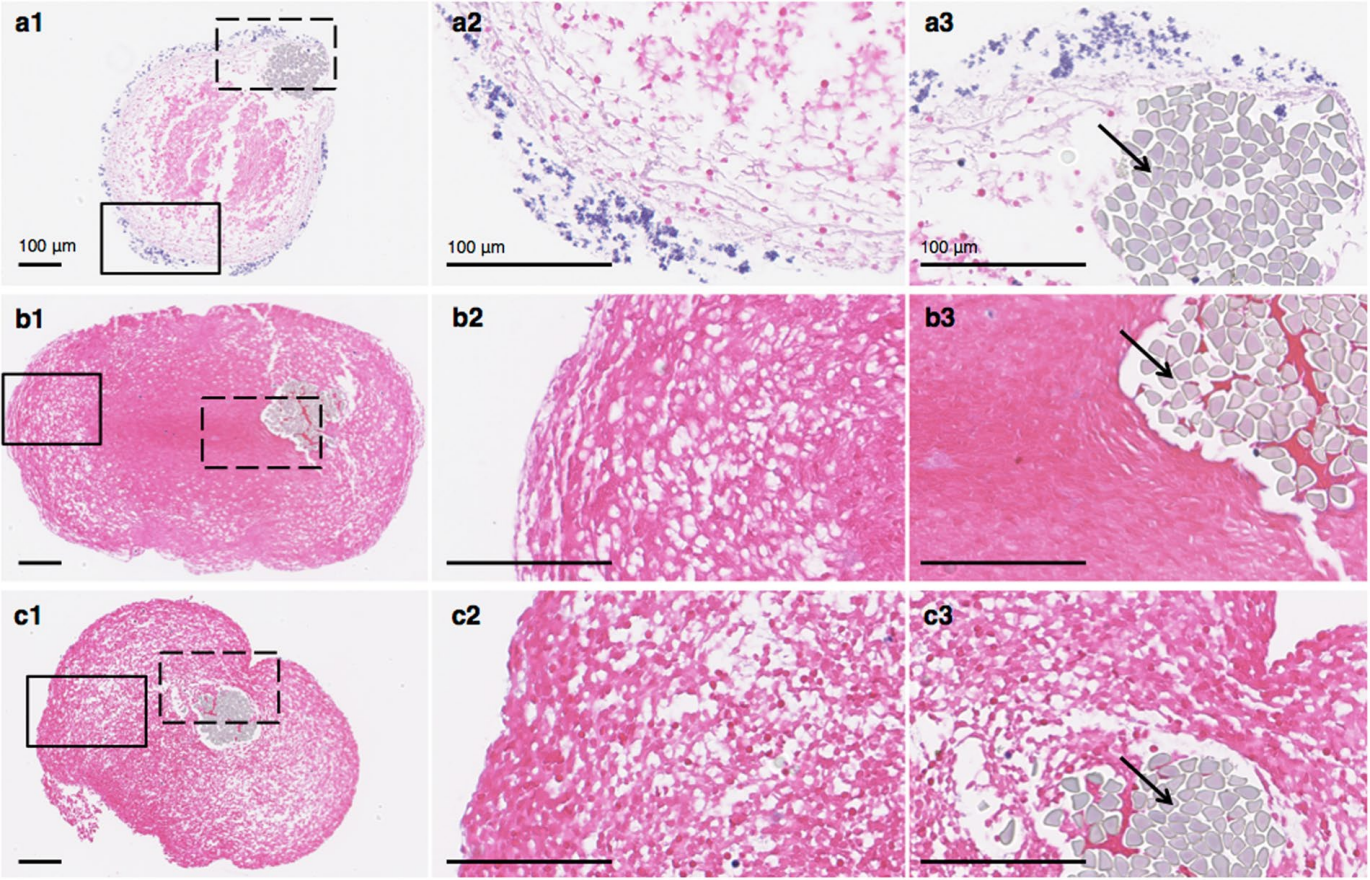
H&E histology of infected and control clot cross-sections. (**a1-3**) Infected clot, (**b1-3**) sterile, retracted clot, and (**c1-3**) sterile, retracted clot incubated 24 hr in sterile hFFP. The boxes with a solid black line in **a1**, **b1**, **c1**, represent the border region of zoomed-in focus for the images to the right (**a2, b2, c2**). The dashed line boxes represent the zoomed-in area of the clot by the suture for the corresponding images to the far right (**a3, b3, c3**). Black arrows indicate a suture thread (light grey). Images (**a1**, **b1**, **c1**) are at 10x magnification, and the rest (**a2-3, b2-3, c2-3**) are at 40x magnification.

When examining the H&E staining that was performed at the same time for all infected and control clots, the infected clots, though from a different batch, had similar morphology (Fig. 2a1–a3) to the previous stained specimens (Fig. 1a). Briefly, a biofilm composed of bacteria encased the fibrin mesh on the outer layer of the clots. Directly below the biofilm was a thick fibrin layer; and below the biofilm layer, the number of erythrocytes present increased towards the predominately erythrocyte core (Fig. 2). The suture (top right in Fig. 2a1–a3) supporting the infected clot was also lined in a fibrin layer and biofilm (Fig. 2a3). The biofilm was heterogeneous with varying thickness. The control clots (Fig. 2b1,c1) were structurally and morphologically different from the infected clots. The sterile retracted clots consisted of a porous perimeter surrounding a dense erythrocyte core (Fig. 2b1). The outer most layer of the perimeter appears to be less porous than the rest of the inner region (Fig. 2b2). This less porous outer layer is more prominent in the sterile retracted clots incubated 24 h in human fresh-frozen plasma (hFFP; Fig. 2c2). This clot was also devoid of the dense core seen in the sterile retracted clot not incubated with plasma. The sutures are eccentric in the infected clot compared to the controls (Fig. 2a3–c3). Additionally, both the infected clot core and the sterile, retracted clot have tear streaks; with multiple found in the infected clot core (Fig. 2a1) and two streaks, one from the left side of the suture to the outside and the other to the right of the suture, of the sterile clot (at 12 o’clock in Fig. 2b1).

### Infected clot degradation as determined by width loss

**Figure 3:**
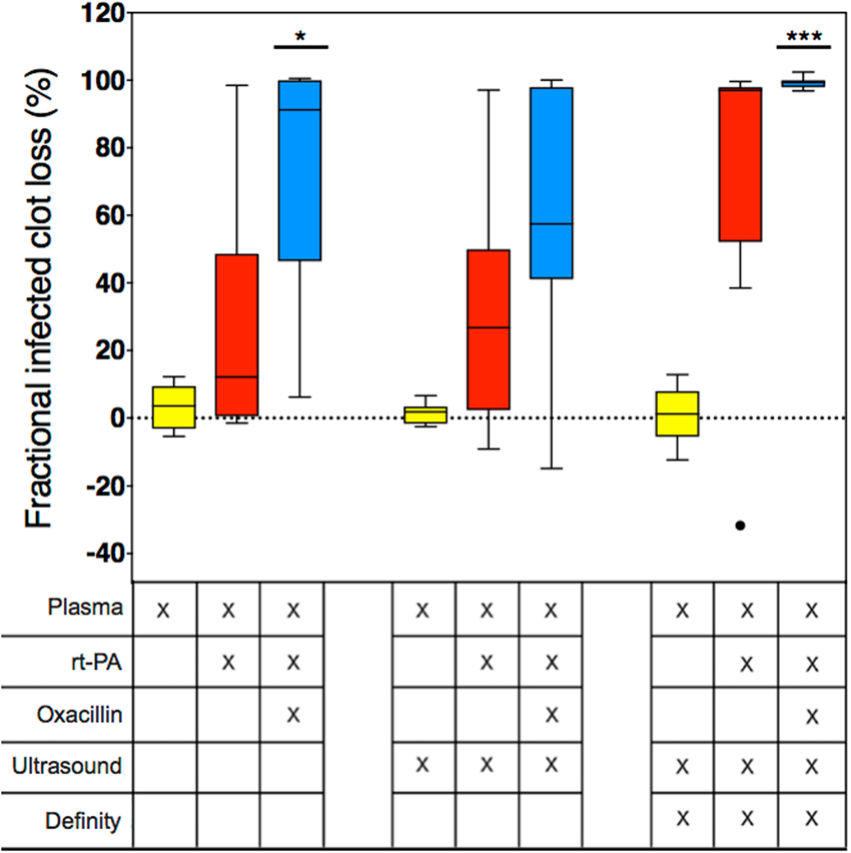
Fractional infected clot loss following different treatments. Boxes represent the interquartile range. The lines within the boxes represent the median and the whiskers indicate 1.5 times the interquartile range. The dot depicts an outlier as determined using the Tukey method. A single asterisk (p < 0.05) or three asterisks (p < 0.001) with a solid line above the boxes represent a statistically significant difference with the plasma alone, the plasma with ultrasound, and the plasma, ultrasound, and Definity treatment groups. The different treatment conditions are given in the table below the graph. N = 9 for all treatments, with the exception of the plasma and ultrasound treated group (n = 10).

In this study, infected clots were subjected to different combinations of therapeutics (oxacillin, rt-PA, intermittent continuous-wave 120-kHz US, and UCA Definity), while subjected to plasma flow under controlled conditions. The infected clots had an average diameter of 442.2 µm ± 47.6 SD with no significant differences between the treatment groups. The fractional infected clot width loss (FICL) over 30 min treatment was used to quantify the extent of degradation, and was computed using custom automated computer image analysis of the time-lapse microscopy images. As seen in Fig. 3, infected clot degradation is reported as the percentage decrease in infected clot diameter at 30 min. A perfusate flow rate of 0.65 mL/min provided minimal degradation of infected clots due to mechanical shear stress and endogenous tissue plasminogen activator from plasma flow alone, thus providing us with a stable, reproducible control. When rt-PA and oxacillin were added to the plasma, infected clot width loss appeared highly variable, ranging from minimal (6.2%) to full clot width loss, i.e. 100% FICL. Similar amounts of degradation as the plasma alone group were seen when US alone under plasma flow was used as treatment. The addition of rt-PA and oxacillin to the perfusate of the US group demonstrated a high level of variability (−14.8–99.9%), similar to the rt-PA and oxacillin without US treatment. The addition of Definity to the plasma and US did not result in large amounts of infected clot degradation (<20%). However, Definity added to the plasma, rt-PA, oxacillin, and US perfusate treatment group resulted in an almost complete loss for all infected clots (99.3 ± 1.7%). This was statistically significantly different than the plasma, US, and Definity treated clots without the rt-PA and oxacillin addition.

### Post-experimental light microscopy

**Figure 4:**
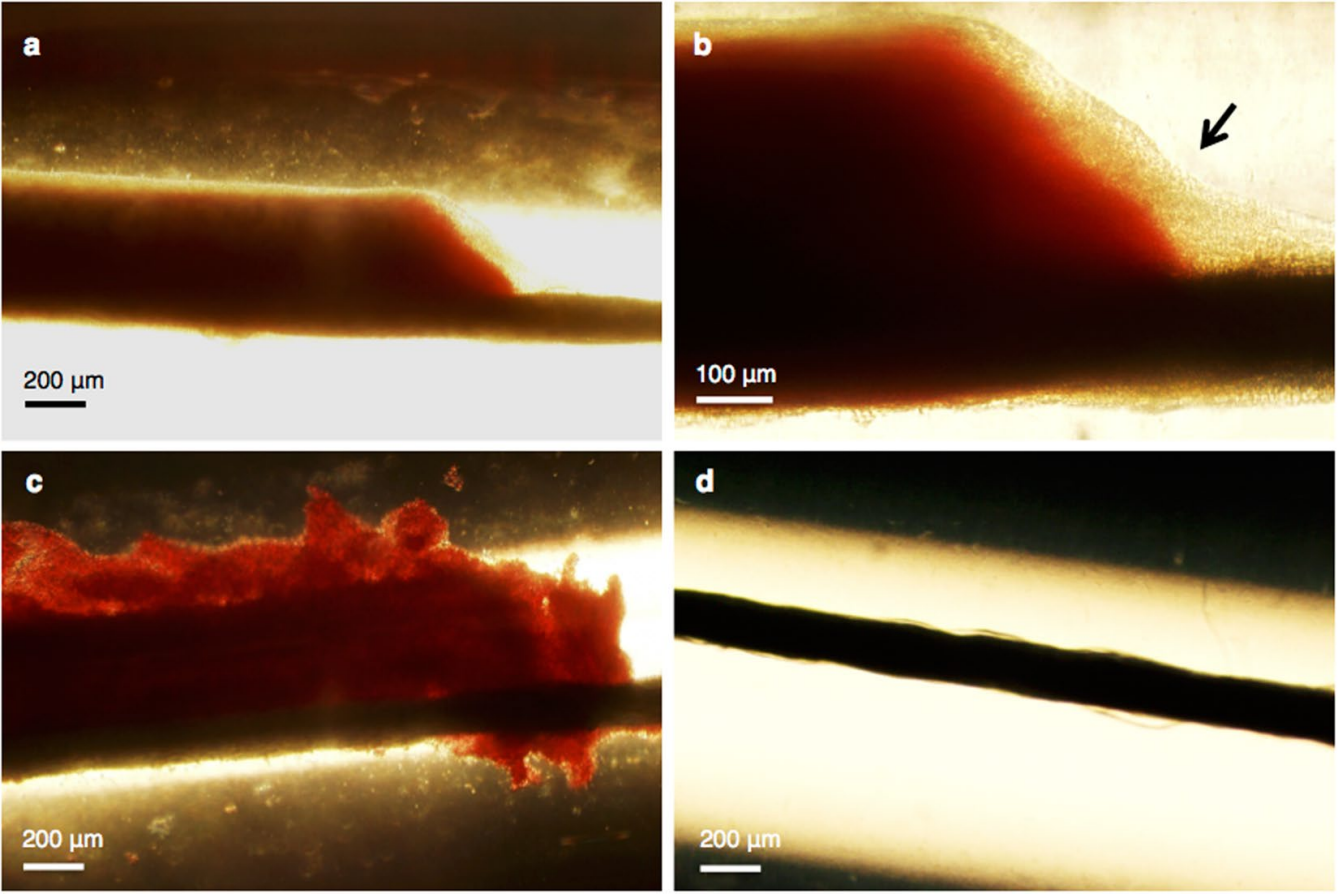
Bright-field light microscopy imaging of infected clots acquired directly following treatment. Infected clot treated only with plasma at 4x (**a**) and at 10x magnification (**b**). The black arrow in image **b** points out the biofilm (beige). (**c**) A plasma, rt-PA, and oxacillin treated infected clot at 4x magnification. (**d**) A plasma, rt-PA, oxacillin, ultrasound, and Definity treated infected clot at 4x magnification. The sutures (black line) are situated at the bottom of the clots, which can be observed to the right of the clot in images **a**–**c**.

In addition to the transverse visualization of the clots during the 30 min time-lapse imaging, a longitudinal perspective was also obtained directly after experiments using light microscopy and a colour camera. Following experiments with plasma alone, the structure of the infected clots appeared intact and adherent to the suture (Fig. 4a). At a higher magnification, the biofilm was visible and observed to be surrounding both the clot (red) and suture (horizontal black line) (Fig. 4b). The images of infected clots treated with plasma alone were consistent with histological findings. Infected clots were composed of a somewhat dense core becoming less dense towards the outer perimeter, and the biofilm as the outermost layer. When infected clots were treated with rt-PA and oxacillin in combination with the plasma, the border of the clot was no longer smooth, but irregular in shape and also appeared less dense (Fig. 4c). This border irregularity was also observed with the monochromatic camera during time-lapse imaging appearing as a rolling-adhesion type motion of fibrin degradation products down the length of the clot. However, the camera and experimental set-up did not allow for capturing the complete length of the clot. After treatment with all therapeutics combined, US exposure, Definity, rt-PA, and oxacillin, revealed a visibly bare suture (Fig. 4d).

### Cavitation detection

**Figure 5:**
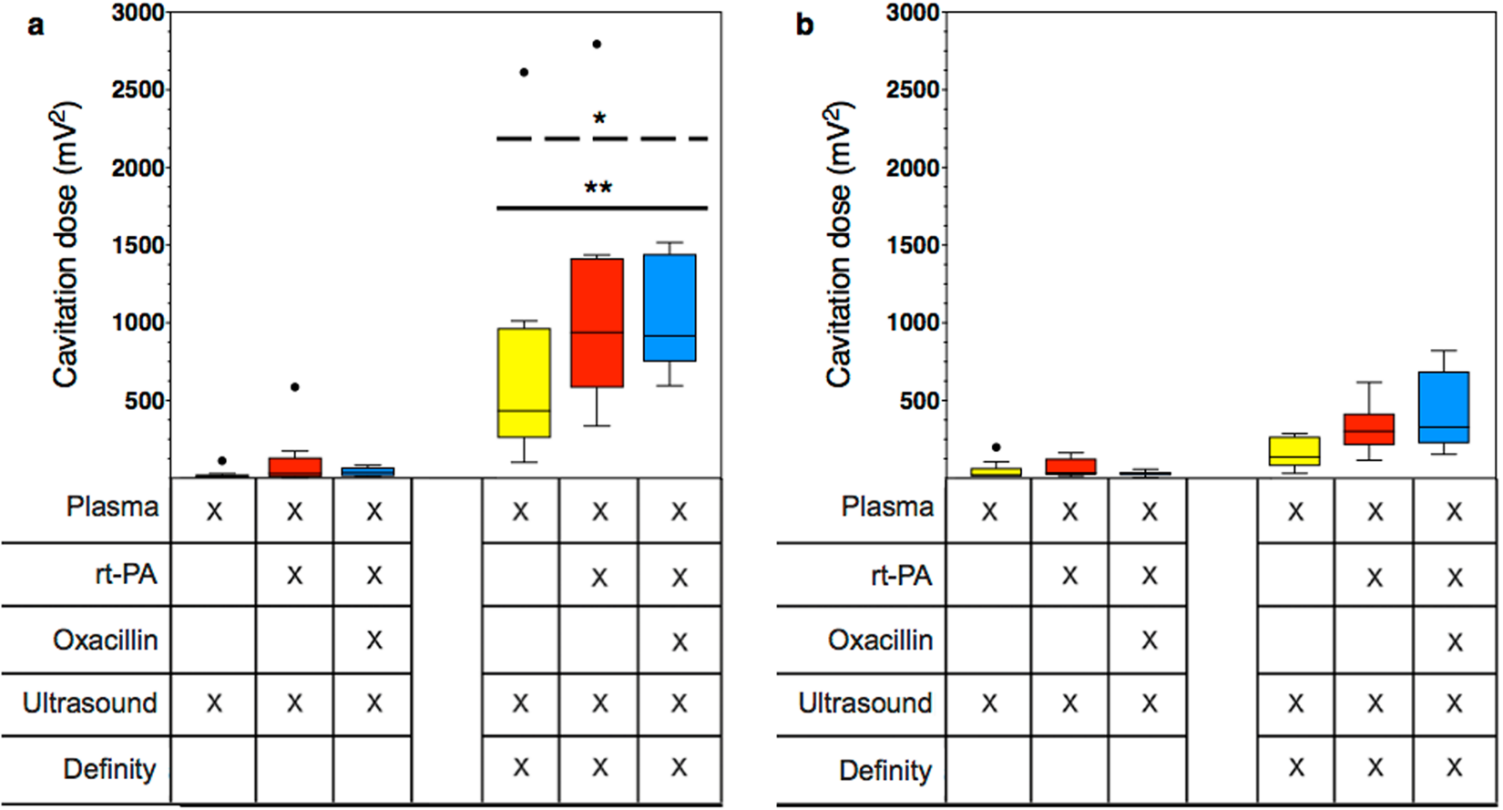
The ultraharmonic (**a**) and broadband (**b**) cavitation energy detected by the passive cavitation detector in response to 120-kHz ultrasound insonification. Boxes represent the interquartile range. The lines within the boxes represent the median and the whiskers indicate 1.5 times the interquartile range. Black circles depict outliers as determined using the Tukey method. A single asterisk (p < 0.05) above a dashed line represents a statistically significant difference between the ultraharmonic and broadband energies of the same treatment group. Two asterisks (p < 0.01) represent a statistically significant difference of ultraharmonic energy between the group and the ultrasound without Definity groups. The different treatment conditions are given in the table below the graph.

Two different types of cavitation energies, ultraharmonic (UH) and broadband (BB), were detected with the passive cavitation detector (PCD), demonstrating the presence of both stable and inertial cavitation. When US without Definity was used in treatment groups, very low levels of cavitation were detected for both types of energy (Fig. 5a,b). The addition of Definity resulted in significant higher levels of UH energy (Fig. 5a), indicating stable cavitation. Additionally, these three treatment groups with Definity exhibited significantly higher UH energy than BB (Fig. 5a,b), which indicates more stable than inertial cavitation. Both UH and BB cavitation was observed to be higher in the Definity, rt-PA and oxacillin treated infected clots, albeit not a significant difference from the other two treatment groups which included Definity.

### Effluent particle size detection

**Figure 6:**
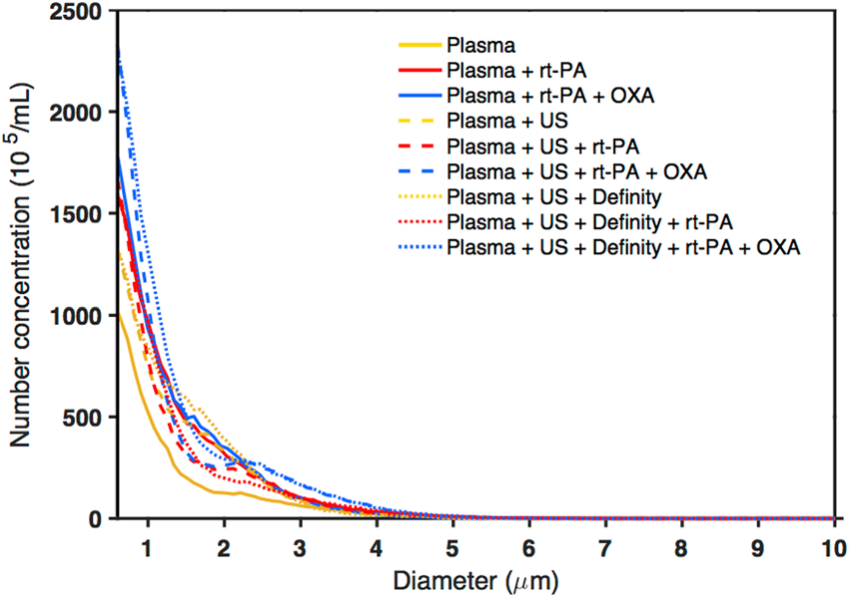
Effluent characterization using particle size measurement with the Coulter counter. Colours and line style represent different treatment groups with yellow indicating no rt-PA or oxacillin, red indicating rt-PA without oxacillin, and blue indicating oxacillin addition (n = 3 per group; line represents the average). Dashed lines indicate treatment with ultrasound; dotted lines indicate treatment with ultrasound and Definity. The number-weighted particle size distribution is shown with the background subtracted. US = ultrasound and OXA = oxacillin.

The number-weighted size distribution of effluent particles, measured directly following treatment, is shown in Fig. 6. The largest particle size detected was 10.4 μm, and 99.6% of the particles were smaller than 5 μm. The plasma treatment alone had the lowest number of particle counts, and the infected clots treated with US, Definity, rt-PA, and oxacillin had the largest amount of counts. Note that the smallest particle-sizing bin of the Coulter counter had a diameter of 0.6 μm.

## Discussion

This proof-of-principle study reports the potential efficacy of sonobactericide, combined with antibiotics and rt-PA, to enhance the treatment of bacterial infected clots in an *in vitro* flow model. In addition, this study also reports on the methods used to create a translatable *in vitro* infected clot model using human and bacterial products, on the basis of the IE isolate, methodology of infected clot, histology, and flow media, to mimic the known early pathogenesis of IE.

To model the *in vivo* situation, a methicillin-susceptible *S. aureus* isolate that originated from an IE patient was used. A clinical IE isolate was chosen for translatability by ensuring the bacteria had the necessary factors to induce IE in a patient, because non-IE originating isolates may lack the bacterial characteristics necessary for proper adherence (e.g. adhesins, fibronectin-, and fibrinogen-binding proteins) to host micro-thrombi to induce IE^33,34^. The isolate’s *spa*-type t021 has previously been isolated from IE^35^. Although we did not directly perform an in-depth virulence factor analysis of this isolate, it has been demonstrated previously that isolates (N = 105) originating from IE patients from different countries all contained the same surface-associated adhesins shown to be important for the invasiveness and development of IE (clfA, clfB, fnbA, and sdrC)^36,37^. Additionally, these factors important for IE development are highly conserved^38^. *S. aureus* is a known producer of coagulase, an enzyme that converts soluble fibrinogen into fibrin^39^. This fibrin scaffold allows the bacteria to encase themselves in a protective structure, consisting of bacterial and host components^40,41^. A marked presence of gram-positive bacterial colonies encapsulated in fibrin is seen in our histology (Figs 1 and 2), which is consistent with that of IE in *in vivo* animal models and human surgically excised valves^5,41–45^.

The progression of the presence of microthrombi on heart valves into IE biofilms is dependent on bacterial adherence to these initial clots^7,41^. Two previous studies developed infected clots as an *in vitro* model of IE, using a mix of human, animal, and synthetic products^46,47^. McGarth *et al*.^46^ suspended bacteria in an eppendorf tube with a fibrin glue recipe consisting of human cryoprecipitate, bovine thrombin, monofilament line, and calcium chloride to create infected clots. Another study reported by Palmer, *et al*.^47^ modified the previous method to include platelets and aprotinin (bovine) solution. Our study used infected retracted human blood clots, and the only synthetic media used was IMDM, which both may allow for bacterial adherence that better mimics the human situation *in vivo*. Additionally, this methodology resembles the early pathogenesis of IE, in which micro-thrombi are formed on heart valves first and then bacteria adhere and subsequently grow into an IE biofilm^1,5,7^. Human cell culture media for growing bacteria have previously been used^48–50^. IMDM was chosen because it emulates the iron-restricted environment *in vivo*. Iron restriction leads to the upregulation of virulence factors which would not be present if grown in a traditional iron rich bacterial growth media^51^.

Histology and live cell imaging revealed that the morphology of infected clots reported in our study (Figs 1a,b and 2) were consistent with the pattern of biofilm growth^6^ and resembled the structure of IE biofilms found in animal and patient samples^5^. Though limited polymorphonuclear (PMN) leukocytes are seen in our histology, neutrophilic inflammation is commonly mentioned in histopathologic findings *in vivo*. However, this inflammation is seen with regards to the valve tissue itself, resulting from endocardial injury^1^. Furthermore, it is well established *in vivo* that biofilm in IE represents a zone of localized agranulocytosis and that it would be rare that PMNs would be able to come into direct contact with the fibrin embedded bacteria^43,44^. This is supported by the clear separation of bacterial colonies and PMNs seen in histologic studies of animal and human IE biofilms^5,41,42,45^. Heterogeneous accumulation of bacteria is evident in the confocal microscopy images of the biofilm (e.g. bottom left corner, Fig. 1c) which is not contiguous in some places. This irregularity could also be due to the friable nature of infected clots after thin sample preparation, which could have been damaged during transport from fluorescent staining to the CLSM system or during the image capturing process.

It is widely known that the efficiency of a drug given intravenously can be affected by binding of the drug to plasma proteins^52^. Our flow model included the interaction of oxacillin with plasma proteins and inhibition of fibrinolysis with plasminogen activator inhibitor (e.g. PAI-1) and antiplasmin^53^ since we used human plasma as our flow media. Additionally, MB oscillations can be affected by the viscosity of the surrounding medium, with damping occurring with increased viscosity^54^. Thus, inclusion of human plasma in our flow studies provided a test of the therapeutic effect of sonobactericide in the presence of a higher viscosity fluid approximating whole blood.

It has been demonstrated that IE biofilms *in vivo* form and generally exist on the low-pressure chamber side of valves^55^. This side of the valves are exposed to normal and regurgitant jet flow, however early IE biofilms are located beside the jet flows where low velocities exist^56^. Additionally, a range of velocities (0 to 45 cm/s over one cardiac cycle) have been reported across the mitral valve^57^, which is a common site of IE. We chose 0.3 cm/s for all experiments in the *in vitro* set-up because 1) this is within range of flow speeds experienced by IE biofilms; and 2) this presents the worst-case scenario for the therapeutic outcome^58–60^. Additionally, the rate of penetration of therapeutics (both rt-PA and the antibiotic), replenishment of microbubbles as cavitation nuclei, and the removal of fibrin degradation products occurs is proportional to the flow conditions^58–60^, meaning the rate is slow under low flow conditions and fast under high flow conditions.

In this study, US and Definity alone did not enhance infected clot size reduction (Fig. 3). There were some clots that showed negative values for infected clot width loss. The response of clots to the thrombolytic and antibiotic may appear as growth, when the infected clot is in the early stages of degradation (Fig. 4c). It is likely that these infected clots could have been effectively lysed provided the treatment time was longer than the 30 min period employed in this study.

Sustained stable cavitation was harnessed in this study, monitored using a PCD detecting UH and BB emissions during 30 min flow experiments. In all cases, we observed significantly more UH than BB cavitation (Fig. 5), indicating more stable than inertial cavitation^29^, which suggests that unwanted bioeffects may be minimized^15^. Paradoxically, stable cavitation, which occurs at a lower acoustic pressure amplitude, has been reported to correlate with enhanced thrombolysis to a greater degree than inertial cavitation in the presence of rt-PA^20^. Although UH was observed when Definity was combined with US, the addition of oxacillin and rt-PA was necessary to achieve infected clot width loss.

The inclusion of rt-PA in combination with oxacillin in our treatment regime likely benefited from this particular lytic’s ability to target fibrin, which is the backbone of IE biofilm^5,61,62^. The absence of large fibrin degradation products liberated during treatment in our study (Fig. 6) suggests minimal risk of embolization from sonobactericide. Nonetheless, filters such as those deployed in vessels downstream of the heart valve during transcatheter aortic valve replacement procedures^63^ could be used with sonobactericide to prevent embolism.

In this study, a 120-kHz frequency was chosen considering that the majority of previous studies for treating bacterial biofilms and clots in the presence of rt-PA have been performed with low frequency US^10–14,21–24,64^. The intermittent exposure scheme has been reported previously for sonothrombolysis^29,65^. However, diagnostic cardiac imaging is typically performed using a 2-MHz centre frequency^66^. Further studies should investigate the feasibility of using dual diagnostic and therapeutic 2-MHz cardiac imaging probes for image-guided treatment of IE. Microbubbles at 1–2 μm in diameter are closer to resonance at 2-MHz than at 120-kHz, therefore lower acoustic pressures could be used to produce sustained stable cavitation, and therefore reduce the likelihood for any negative bioeffects to surrounding tissues.

The host immune system response, which includes inflammatory processes, is not adequately represented in our *in vitro* IE biofilm model. Another limitation is that dynamic flow was not present during the formation of the infected clot. Biofilms developed in a mostly static condition *vs*. a dynamic condition can lack robustness^67^. Additionally, only one isolate was tested in this study. More isolates must be tested in future studies to determine the ability of sonobactericide to treat other species or other isolates of *S. aureus* causing IE. Finally, this study did not investigate the direct bactericidal capability of sonobactericide in combination with antibiotics and thrombolytics, which is important in understanding the true potential of this treatment. However, it is known that bacteria released from mature biofilms become metabolically active and are thus susceptible to antibiotic treatment in the blood stream^6,8^. Freed bacteria as a result of sonobactericide should be evaluated in future studies.

Traditionally, IE is considered a contraindication for thrombolytic treatment in adults^31,32^. However, is has been shown to be effective in paediatric patients^25–28^. Unlike other thrombolytics, rt-PA has a high affinity to fibrin and thus provides a more local activation of fibrin-bound plasminogen, therefore decreasing the risk of negative effects due to systemic plasminogen cleavage^61,62^. Localized delivery of rt-PA to IE biofilms using echogenic liposomes loaded with rt-PA in combination with an antibiotic could also be a promising strategy to reduce off-target effects^65^.

Lastly, studies in an IE animal model will be necessary to assess the efficacy of sonobactericide *in vivo*. Nonetheless, the work reported in this paper represents the first time that sonobactericide has been investigated as a possible therapeutic option for infective endocarditis. Accordingly, our primary goal was to determine suitable treatment conditions and to understand if this approach has potential in a tightly controlled setting. The rationale for using the *in vitro* model was to allow for precise cavitation monitoring, complete dose control, and to minimize human and microbiological variability in the infected clot. Further advantage of an *in vitro* model such as ours is constant visibility of the infected clot throughout treatment with time-lapse microscopy. Nevertheless, it has been previously demonstrated that the results of *in vitro* simulated endocarditis biofilms (infected clots) are comparable to the *in vivo* rabbit model of endocarditis for the study of fluoroquinolone efficacy, to include pharmacokinetics^68^.

## Conclusion

In this proof-of-principle study, infected clots were developed as a translatable model of IE biofilm and the efficacy of sonobactericide, the use of US and an UCA in combination with or without an antibiotic and thrombolytic was evaluated *in vitro* under flow. Histology and confocal imaging revealed that the infected clot model resembled a clinical IE biofilm, especially for early pathogenesis. Infected clots exposed to the combination of oxacillin, rt-PA, ultrasound, and Definity achieved 99.3 ± 1.7% fractional infected clot loss, which was greater than the other treatment arms. These results suggest that sonobactericide may have potential as an adjunctive therapy for IE.

## Materials and Methods

### Bacterial isolate

The *S. aureus* (JC01-2016) used in this study was an anonymized, de-identified strain isolated from an IE patient at Cincinnati Children’s Hospital Medical Center, collected in accordance with guidance from the Institutional Review Board. According to institutional review board policy, anonymized, de-identified bacterial isolates such as JC01-2016 are considered Non-Human Subject Research and do not require informed consent. All experimental protocols in this study were approved by the University of Cincinnati institutional review board, and all methods were carried out in accordance with relevant guidelines and regulations. Overnight cultures of the isolate in tryptic soy broth (MP Biomedicals, USA) were added to DMSO (15%; Fisher Chemical, USA) and stored at −80 °C. All overnight cultures from frozen stocks were streaked on tryptic soy agar (TSA; MP Biomedicals, USA) and incubated at 37 °C.

Bacterial chromosomal DNA was isolated from a single colony using DNeasy Ultraclean Microbial kit (MoBio Laboratories, California, USA). Amplification and sequencing of the polymorphic X region of the Protein A gene was performed using spa1095F (5′-AGACGATCCTTCGGTGAGC-3′) and spa1517R (5′-GCTTTTGCAATGTCATTTACTG-3′) (Integrated DNA Technologies Inc., Iowa, USA)^69–71^. Amplification and sequencing was verified using primers spa1113F (5′-TAAAGACGATCCTTCGGTGAGC-3′) and spa1514R (5′-CAGCAGTAGTGCCGTTTGCTT-3′)^69^. PCR amplification (Mastercycler pro S, Eppendorf, New York, USA) was accomplished using 22 µl of sterile water, 1 µl of forward primer, 1 µl of reverse primer, 2 µl of isolated chromosomal DNA, and 24 µl of MidSci Taq Plus Mastermix with red tracer dye (Midsci, Missouri, USA). Thermocycler parameters were set as previously described^71^. Sequences were analysed for polymorphic X region repeats and matched to repeat succession sequences provided by Ridom SpaServer Database (http://spa.ridom.de) to determine the *spa*-type^70^.

### Antibiotic susceptibility testing and growth curve assessment

Oxacillin (28221; Sigma-Aldrich, Missouri, USA) susceptibility was determined (n = 3) using the agar dilution method on Mueller-Hinton agar (Sigma-Aldrich, Missouri, USA), supplemented with 2% NaCl^72^. *S. epidermidis* ATCC 35984 (RP62a) was used only as a quality control for antibiotic susceptibility testing. Bacterial growth curves (n = 3) with an initial OD_600nm_ of 0.05 were produced as described by Harris *et al*.^73^ with the exceptions of IMDM (containing no phenol red; Gibco, USA) instead of brain heart infusion medium, and samples of 3 mL were measured every 30 min for 9 h and a final sample measured at 24 h.

### *S. aureus* inoculum preparation for infected blood clot formation

To prepare the *S. aureus* isolate for inoculation of blood clots, early exponential growth phase grown bacteria were generated following the same protocol for determining growth curves. Cultures with a starting OD_600nm_ of 0.05 were incubated at 37 °C in IMDM. After 3.5–4 h of growth the *S. aureus* isolate was in the mid-exponential growth phase, and a 1.5 mL sample at an OD_600nm_ of 1 (approximately 1 × 10^9^ CFU/mL) was obtained. The bacteria were grown to mid-exponential phase because this is when the expression of surface-associated adhesins generally occurs to facilitate initial colonization^74^. This bacterial suspension was kept at 37 °C and subsequently used for inoculation within fifteen minutes of preparation.

### Infected clot formation as model for IE

To produce the infected clots to resemble the *in vivo* early pathogenesis of IE, first human whole blood clots were created around silk sutures as previously described by Bader *et al*.^29^ with the exceptions of complete experimental sterility and suture brand and size. Specifically, 9 cm sections of 6–0 silk sutures (1639G; PERMA-HAND, Ethicon Inc., USA) were threaded into 2.5 cm long borosilicate glass capillaries (1.12 mm inner diameter, World Precision Instruments Inc., USA). These capillaries were subsequently placed inside borosilicate glass culture tubes (10 mm diameter x 75 mm height, VWR International, USA). With University of Cincinnati institutional review board approval and written informed consent, venous whole blood was drawn from five healthy volunteers. Five hundred μL aliquots were transferred into each tube, and by capillary action blood was drawn into the capillaries. The glass tubes were incubated for 3 h at 37 °C to allow the blood to clot around the suture, followed by refrigeration at 4 °C for a minimum of three days to promote clot retraction. These retracted clots are stable and resistant to complete rt-PA thrombolysis^75^.

After retraction, the clots were incubated at 37 °C for 30 min. Aliquots of 30 minutes pre-warmed sterile hFFP (3.325 mL; Hoxworth Blood Centre, Cincinnati, Ohio, USA) were placed into borosilicate glass culture tubes, and inoculated with 175 μL of the prepared bacterial inoculum. The retracted clots were removed from the glass capillaries and carefully placed in the inoculated hFFP. To ensure the bacterial inoculate had reached all surfaces of the clot, the glass tubes were gently inverted approximately 8 times. Subsequently, the glass tubes were incubated for 24–30 h at 37 °C, and reinverted every hour for approximately the first five hours.

Before placement in the *in vitro* flow system, infected clots were washed three times with PBS to remove any planktonic bacteria. Additionally, for histological analysis, two additional clot controls were used: sterile retracted clots alone; and sterile retracted clots following infected clot protocol, with the exception of using no bacteria and thus incubating for 24–30 h in hFFP.

### Histology

Clots were fixated in 10% neutral buffered formalin. Following fixation, clots were transferred to a tissue cassette containing a foam biopsy pad (first set specimens), or without a foam pad (2^nd^ set specimens). These specimens were processed, embedded in paraffin, sliced (4 μm), mounted on a microscopy slide, and stained with either H&E or CV for cross-sectional analysis. Images were either captured using a camera (Digital Sight DS-Ri1, Nikon, Japan) mounted on a microscope (BX51, Olympus Inc., USA) with imaging software (NIS-Elements Basic Research, Nikon) for the first set of clot specimens, or with a virtual microscope digital slide scanner (C9600; NanoZoomer 2.0HT, Hamamatsu Photonics, Japan) and corresponding digital pathology viewing software (U12388–01; NDP.view2, Hamamatasu, Japan) for the second set of specimens.

### CLSM

Individual infected clots were placed in a sterile chambered #1.5 polymer coverslip (80286; μ-slide 2 Well, ibiTreat, ibidi GmbH, Germany). Following the manufacturer’s instructions, the LIVE/DEAD BacLight bacterial viability fluorescence kit (L7012, Molecular Probes, Oregon, USA) was used. After incubation, the chamber was flooded with PBS, and then placed in the CLSM holder, where it was observed with an upright Nikon FN1 microscope attached to a Nikon A1R confocal system. A 25 × long working distance objective (CFI Apo LWD NA 1.10 water-immersion) was used.

### Experimental set-up

An *in vitro* flow model, depicted in Supplementary Fig. S1 online was used, which has been described in detail previously^29^. Using a syringe pump in withdrawal mode (Model 44, Harvard Apparatus, Massachusetts, USA), the perfusate flow rate was maintained at 0.65 mL/min (0.3 cm/s), consistent with previously reported sonothrombolysis studies using the same *in vitro* flow set-up^29^ and coinciding with slow blood flow eddies experienced by IE biofilms^55,56^.

The infected clots, along with the perfusate, were insonated with a custom-designed, unfocused, 120-kHz US transducer (60 mm diameter aperture; 5 cm distance to clot; Sonic Concepts, Washinton, USA). Distances for both the passive cavitation detector and the 120-kHz transducers to the clot was 5 cm^76^. Using a function generator (33250 A; Agilent Technologies Inc., California, USA) and power amplifier (1040 L; ENI, New York, USA), the transducer was excited at its resonant frequency (120-kHz). A custom-built impedance matching network (Sonic Concepts Inc., Washington, USA) was used to maximize power transfer to the transducer^29^. The acoustic field was measured and *in situ* acoustic pressure calibrated along the clot using a 0.5 mm hydrophone (TC 4038; Teledyne Reason Inc, California, USA) mounted on a computer-controlled three-axis positioner (NF-90; Velmex Inc., New York, USA)^29^.

A single element acoustic passive cavitation detector (PCD; circular aperture diameter 19 mm; 5 cm distance to clot; 2.25-MHz centre frequency; −6 dB two-way bandwidth of 0.98-MHz; 595516 C; Picker Roentgen GmbH, Espelkamp, Germany) was aligned with the infected clot and used to monitor cavitation activity. As reported previously, ultraharmonic (UH) and broadband (BB) emissions were employed to detect stable and inertial cavitation, respectively^29^. To remove any noise from radiofrequency interference, the received signal from the PCD was filtered by a 10-MHz low-pass filter (J73 E, TTE, California, USA), and amplified with a wideband low-noise amplifier (CLC100, Cadeka Microcircuits, Colorado, USA). The signal was digitized (10 ms duration, 31.25-MHz sampling frequency), and the power spectrum computed in MATLAB (The Mathworks, Massachusetts, USA). To compute the UH energy, UH bands of the power spectrum between 250-kHz and 1-MHz were summed over a 2-kHz bandwidth centred around each UH frequency^29^. The BB energy was computed by summing BB emissions between 250-kHz and 1-MHz in 4-kHz bands centred at each UH band, ± 10-kHz and ± 30-kHz respectively^29^.

### Experimental procedure

For each *in vitro* flow experiment, 30 mL of hFFP was placed in a 500 mL beaker for 2 h to reach gas equilibrium at 37 °C. An infected clot was carefully mounted inside the glass capillary, connected to the flow system, and placed at the bottom of the 37 °C water tank over the microscope objective. The focus of the PCD was aligned with the capillary and the 120-kHz therapeutic acoustic beam, and infected clots were treated for 30 min, which is the half-life of oxacillin in the body^77^. This time has also been reported for sonothrombolysis experiments^29,65^. A pulsed US exposure scheme, shown to promote UH emissions, was used as described by Bader *et al*.^29^. Definity and perfusate were insonated for a period of 50 s at a peak-to-peak pressure of 0.44 MPa. This was directly followed by a 30 s quiescent period to allow a fresh influx of UCA to fill the glass capillary. Acoustic emissions recorded by the PCD were acquired at a rate of 2.33-Hz (0.43 s inter-frame time)^29^. This scheme was repeated in intermittent fashion over the 30 min treatment time.

For infected clots, 9 different experimental flow exposures were included: (1) plasma alone; (2) plasma and the US exposure scheme; (3) plasma, US exposure, and Definity; (4) plasma and rt-PA; (5) plasma, rt-PA, and US exposure; and (6) plasma, rt-PA, US exposure, and Definity; (7) plasma, oxacillin, and rt-PA; (8) plasma, oxacillin, rt-PA, and US exposure; and (9) plasma, oxacillin, rt-PA, US exposure, and Definity. Definity (Lantheus Medical Imaging, North Billerica, Massachusetts, USA)^78^ vials were activated according to the instructions of the manufacture and diluted to a concentration of 2 μL/mL (2 × 10^7^ MBs/mL), the infusion dose previously used for sonothrombolysis^29^. For oxacillin, 172 μg/mL was chosen because this is the peak serum concentration of the antibiotic therapeutic dose in humans treated with IE^79,80^. For all experiments with rt-PA, 3.15 μg/mL was used, which is within the therapeutic dose range for thrombolysis interventions^81,82^. At least 9 experiments were performed for a given treatment, using blood from 5 donors (total of 82 experimental infected clots).

### Calculation of infected clot degradation metrics

To quantify instantaneous infected clot degradation from the images recorded with the CCD camera, an edge-detection and tracking analysis script in MATLAB was used as described previously^29,65^. Briefly, the initial infected clot width (*ICW*_*i*_) at 0 min and the final infected clot width (*ICW*_*f*_) at the completion of the 30 min experiment were used to determine FICL using the following equation:

1 $$FICL=\frac{\,IC{W}_{i}\,-\,IC{W}_{f}}{IC{W}_{i}}\times {100} \% .$$

The width of the infected clot was defined as the distance between the edges for each row of the image, minus the width of the suture.

### Post-experiment bright-field light microscopy

Following *in vitro* flow experiments of plasma alone and the combination of plasma, thrombolytic, and antibiotic, the flow system was left intact and placed on top of an inverted microscope (IX71, Olympus Inc.). Images were obtained using a 12-bit CCD camera (Retiga-EXi, Q-imaging, British Columbia, Canada) equipped to the microscope in order to obtain additional qualitative information (e.g. colour, larger field-of-view) about clots than that inferred from time-lapse imaging.

### Effluent characterization

Effluent samples from the *in vitro* flow experiments were measured directly following the 30 min protocol using a Coulter Counter (Multisizer 4, 30-μm aperture, Beckman Coulter, California, USA) to quantify the size distribution (600 nm–18 μm) of debris, cells, and cell aggregates. This measurement was completed three times for each experimental type, with five replicates per treatment.

### Statistical analysis

Data were statistically analysed using GraphPad Prism 7 (GraphPad Software Inc, California, USA) with a significance level of p < 0.05. To analyse the FICL difference of means among the experimental exposures for infected clots, the Kruskal-Wallis test was used. Additionally, post-hoc testing was performed using Dunn’s non-parametric pairwise multiple comparison test. The UH and BB cavitation energy difference of means was analysed using a two-way ANOVA with post-hoc analysis using Tukey’s multiple comparisons test. For both the cavitation energy and FICL analyses, the Tukey method was used to calculate and report medians and interquartile ranges.

### Data availability statement

The data generated during and/or analysed during the current study are available from the corresponding author upon reasonable request.

## Electronic supplementary material

Supplementary Information
