# Supplementary material for: An in vitro proof-of-principle study of sonobactericide
Source: Sci Rep. 2018 Feb 21;8:3411. doi: 10.1038/s41598-018-21648-8 (PMC5821825; doi:10.1038/s41598-018-21648-8)
Supplement: Supplementary file 1 — Supplementary Information [file 41598_2018_21648_MOESM1_ESM.doc]

Supplementary Information for

**An *in vitro* proof-of-principle study of sonobactericide**

*Kirby R. Lattwein^1,2,3^, Himanshu Shekhar^2^, Willem J.B. van Wamel^3^, Tammy Gonzalez^4^, Andrew B. Herr^4^, Christy K. Holland^2^, and Klazina Kooiman^1^

^*1^Department of Biomedical Engineering, Thoraxcenter, Erasmus MC, Room Ee2302, P.O. Box 2040, 3000 CA, Rotterdam, the Netherlands

^2^Department of Internal Medicine, Division of Cardiovascular Health and Disease, University of Cincinnati, Cincinnati, Ohio, USA

^3^Department of Medical Microbiology and Infectious Diseases, Erasmus MC, Rotterdam, the Netherlands

^4^Cincinnati Children’s Hospital Medical Center, Division of Immunobiology, Center for Systems Immunology, and Division of Infectious Diseases, Cincinnati, Ohio, USA

***Correspondence:** Kirby R. Lattwein

**E-mail address:** k.lattwein@erasmusmc.nl

**Work telephone number:** 0031 10 7044633

**Work fax number:** 0031 10 7044720


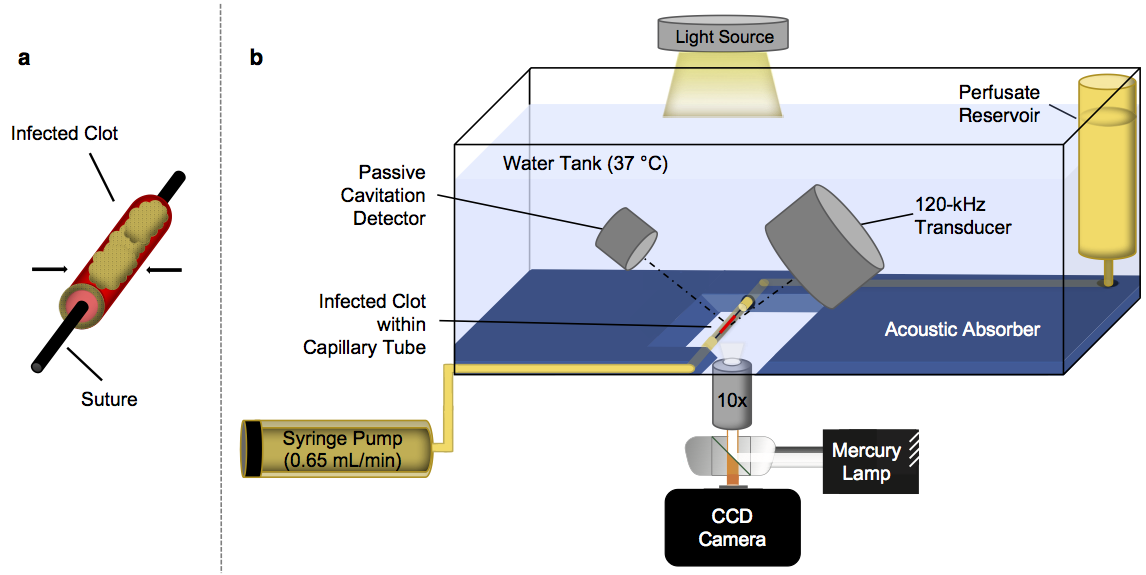


**Figure S1. Experimental set-up.** (a) Illustration of the infected clot model (442.2 μm ± 47.6 SD in width), where the infected clot composed of bacteria (yellow) and clot (red) is produced on a suture (black). (b) Schematic depicting the combined time-lapse microscopy, acoustical field set-up, and flow system to study the treatment of infected clot on a suture *in vitro* (not drawn to scale). For clarity purposes, only the bottom wall of the tank in the schematic is lined with acoustic absorber, while in reality all walls were lined. Distances for both the passive cavitation detector and the 120-kHz transducers to the clot was 5 cm. The perfusate reservoir contained plasma alone, or plasma and therapeutics.
